# Measurement of excitation-inhibition ratio in autism spectrum disorder using critical brain dynamics

**DOI:** 10.1038/s41598-020-65500-4

**Published:** 2020-06-08

**Authors:** Hilgo Bruining, Richard Hardstone, Erika L. Juarez-Martinez, Jan Sprengers, Arthur-Ervin Avramiea, Sonja Simpraga, Simon J. Houtman, Simon-Shlomo Poil, Eva Dallares, Satu Palva, Bob Oranje, J. Matias Palva, Huibert D. Mansvelder, Klaus Linkenkaer-Hansen

**Affiliations:** 1 Department of Child and Adolescent Psychiatry, Amsterdam UMC, University of Amsterdam, Meibergdreef 5, 1105 AZ Amsterdam, The Netherlands; 2 Department of Psychiatry, UMC Utrecht Brain Center, University Medical Centre Utrecht, Heidelberglaan 100, 3584CG, Utrecht, The Netherlands; 3 Department of Integrative Neurophysiology, Center for Neurogenomics and Cognitive Research (CNCR), Amsterdam Neuroscience, VU University Amsterdam, 1081 HV, Amsterdam, The Netherlands; 4 Neuroscience Institute, New York University School of Medicine, 435 East 30th Street, New York, NY 10016 USA; 5 NBT Analytics BV, Amsterdam, The Netherlands; 6 Neuroscience Center, Helsinki Institute for Life Sciences, University of Helsinki, FIN-00014 Helsinki, Finland; 7 BioMag Laboratory, HUS Medical Imaging Center, Helsinki University Central Hospital, FIN-00029 Hus, Finland

**Keywords:** Computational biology and bioinformatics, Neuroscience, Biomarkers, Medical research

## Abstract

Balance between excitation (E) and inhibition (I) is a key principle for neuronal network organization and information processing. Consistent with this notion, excitation-inhibition imbalances are considered a pathophysiological mechanism in many brain disorders including autism spectrum disorder (ASD). However, methods to measure E/I ratios in human brain networks are lacking. Here, we present a method to quantify a functional E/I ratio (*fE*/*I*) from neuronal oscillations, and validate it in healthy subjects and children with ASD. We define structural E/I ratio in an *in silico* neuronal network, investigate how it relates to power and long-range temporal correlations (LRTC) of the network’s activity, and use these relationships to design the *fE*/*I* algorithm. Application of this algorithm to the EEGs of healthy adults showed that *fE*/*I* is balanced at the population level and is decreased through GABAergic enforcement. In children with ASD, we observed larger *fE*/*I* variability and stronger LRTC compared to typically developing children (TDC). Interestingly, visual grading for EEG abnormalities that are thought to reflect E/I imbalances revealed elevated *fE*/*I* and LRTC in ASD children with normal EEG compared to TDC or ASD with abnormal EEG. We speculate that our approach will help understand physiological heterogeneity also in other brain disorders.

## Introduction

Excitatory (E) and inhibitory (I) systems are critical for regulating the flow of information in the brain. Without narrow control over the E/I ratio, runaway excitation or quiescence would occur, impeding adequate information processing^1,2^. In clinical terms, disruption of E/I balance has become a dominant theory on the pathogenesis of various neurodevelopmental disorders, and perhaps most explicitly in autism spectrum disorder (ASD)^3–6^. The nature, however, of implicated E/I imbalances on ASD is diverse, ranging from molecular changes to altered neuronal circuits^3,7,8^ and has been hypothesized to explain some of the variability in treatment responses^7^. E/I ratio may be regulated at the level of synaptic currents and network connectivity^2,4,9,10^ and both levels affect the dynamics of ongoing network activity^11–16^. Thus, it may be possible to derive a quantitative measure of E/I ratio from ongoing brain activity, e.g., as measured with electroencephalography (EEG). Such a measure would allow testing hypotheses about the functional role of E/I ratio^8^, could enable physiological stratification within neurodevelopmental disorders and facilitate personalized application of E/I-modulating therapies^3,7,17^.

The theory of critical brain dynamics links E/I balance to the scale-free statistical character of network activity^18–25^. Specifically, neuronal networks exhibit scale-free spatial and long-range temporal correlations of activity patterns when they operate near the critical point, poised between a low-activity sub-critical phase—which occurs when there is excessive net inhibition—and a relentlessly active super-critical phase associated with excessive net excitation^18,26^. Of note, even though the spectral power of activity increases with increasing excitation it is not possible to infer the transition point where excitation and inhibition in the network are in balance from power analysis alone^25^. Hence, it is plausible that combining two properties of neuronal network activity—the spectral power and long-range temporal correlations—can lead to an estimate of E/I ratio. Here, we develop this idea using a computational model of neuronal network oscillations and test the algorithm using EEG datasets from healthy controls and children with ASD.

## Results

### Development of an algorithm to estimate E/I ratio from neuronal oscillations

To develop an algorithm for estimating E/I ratio, we used an extended version of the Critical Oscillations (CROS) *in silico* model of ongoing neuronal activity^25^ (Methods). It has been shown previously that the activity generated by the model reproduces the statistics of amplitude fluctuations of neuronal oscillations in humans^27,28^ and the spatial spreading of activity in cortical slices^23^ when there is a certain balance between the number of excitatory and inhibitory connections in neuronal networks^25^. Therefore, we introduce here a definition of a network-level structural E/I ratio and use this parameter to control the activity in the model with the aim to develop a functional measure of excitation/inhibition ratio.

**Figure 1:**
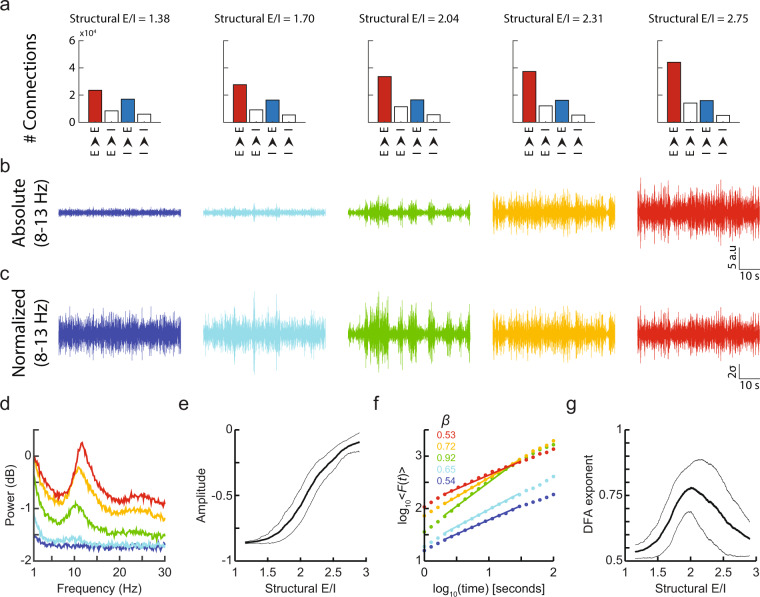
Amplitude and long-range temporal correlations of oscillations strongly depend on structural E/I. (**a**) Number of connections between neuron types for five different networks with increasing structural E/I. (**b**) Network activity filtered in the 8–13 Hz band shows increasing oscillation amplitude with increasing structural E/I. (**c**) Z-scored activity of (b) shows differing temporal structure of oscillation amplitude for balanced and unbalanced networks. (**d**) Power spectrum for networks in (b). (**e**) Spectral amplitude in the 8–13 Hz band increases with structural E/I. Running mean amplitude values of 300 networks (*thick line*) +/− 1 standard deviation (*thin lines*). (**f**) Detrended fluctuation analysis (DFA) applied to the amplitude envelope of 8–13 Hz filtered signals in b shows increasing scaling exponents for balanced (*green*) compared to unbalanced networks (*blue*, *red*). (**g**) DFA exponents show an inverse u-shaped relationship with structural E/I.

We define the network’s structural E/I ratio (*sE*/*I*) as the number of excitatory-to-excitatory synapses divided by the number of inhibitory-to-excitatory synapses. The assemblies in the CROS model produce activity across a wide range of frequencies and prominent oscillations in the 8–13 Hz range for most combinations of excitatory and inhibitory connectivity (Fig. 1d)^25^. Examining the spectral amplitude of these oscillations, we found a clear increase in amplitude with increasing *sE*/*I* (Fig. 1a,b,d,e). The temporal structure of the network oscillations also varied with changes in *sE*/*I* with notably larger temporal heterogeneity at an intermediate value of *sE*/*I* (Fig. 1b,c). To quantify this effect, we applied detrended fluctuation analysis (DFA) to the amplitude envelope of the alpha oscillations to obtain the DFA exponent, *β*, which is a measure of long-range temporal correlations (LRTC) and widely used as an index of critical oscillation dynamics^21,27^. DFA exponents significantly above 0.5 and below 1.0 indicate LRTC of a power-law form. DFA of network signals from the CROS model indeed revealed significant LRTC (Fig. 1c,f). Importantly, these LRTC showed an inverse U-shaped relation with *sE*/*I* so that their scaling exponents peaked when excitation and inhibition were in a certain balance to one another (Fig. 1g).

**Figure 2:**
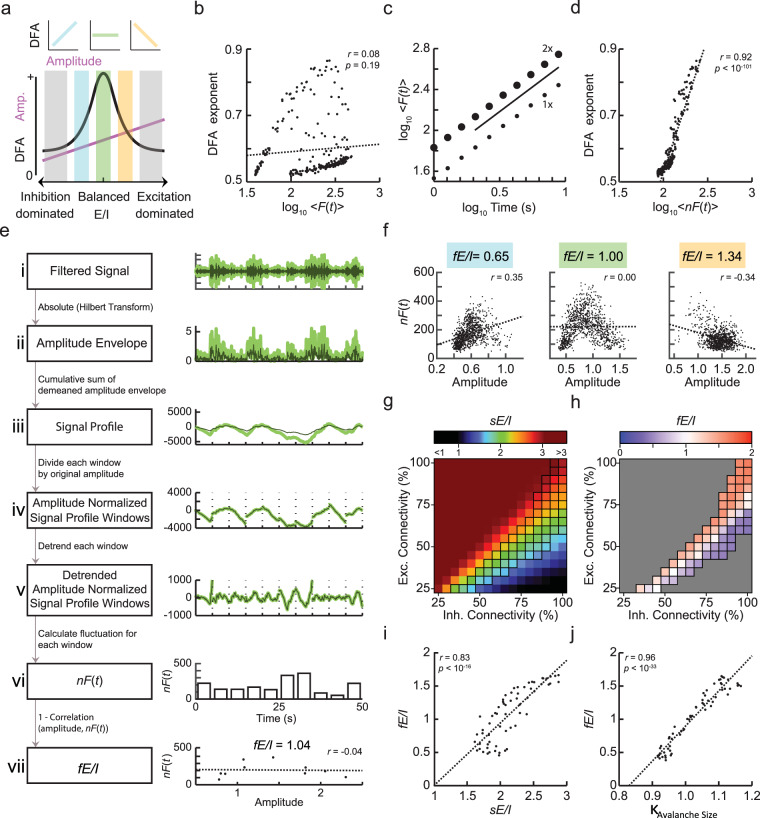
Joint fluctuations in the amplitude and scaling of oscillations enable estimation of the excitation-inhibition ratio of a neuronal network. (**a**) Similar LRTC (quantified by DFA) can be produced by two networks with different *sE*/*I*. In the blue area, increasing LRTC correspond to increasing amplitude of oscillations, in the orange area decreasing LRTC correspond to increasing amplitude of oscillations. (**b**) The mean fluctuation (log_10_ < *F*(*t*)>) for a window size of 5 seconds is a poor predictor of the DFA exponent. Each dot represents one network realization simulated for 1000 seconds, and the mean fluctuation and the DFA exponent are derived from the entire signal. (**c**) Mean fluctuation values (log_10_ < *F*(*t*)>) for a window-size scale proportionally with signal magnitude and with LRTC. Therefore, if we normalize the amplitude of the signal for a certain window size then the fluctuation in that window size would be an estimate of the DFA exponent. (**d**) After normalizing the signal profile windows, the DFA exponents can be estimated by their mean fluctuation, log_10_ < *nF*(*t*)> (shown for a window size of 5 seconds). Each value represents the average of 20 networks for each combination of excitatory and inhibitory connectivity parameters. (**e**) Method to calculate *fE*/*I*. (*i*) The model signal filtered in the alpha range (*dark thin green*), and the same signal with double the magnitude (*thick light green*). (*ii*) Amplitude envelope of the two signals. (*iii*) Signal-profile deviations are greater for the larger-magnitude signal (*light green*). (*iv*) Dividing each window by its original mean amplitude, removes any effect of original magnitude on the signal profile. (*v*) Subtract the linear trend. (*vi*) Calculate the standard deviation for each window to get the normalized fluctuation function, *nF*(*t*). (*vii*) *fE*/*I* is calculated by correlating the mean windowed amplitudes calculated from *ii* and the windowed *nF*(*t*) calculated in (*vi*). (**f**) Correlation between windowed values for *nF*(*t*) and amplitude is used to create the estimate of *fE/I*, shown for three example networks with low (1.82, *left*), medium (2.05, *middle*), and high (2.25, *right*) *sE*/*I*, respectively. (**g**) The *sE*/*I* index is shown in color scale for the phase space of inhibitory and excitatory connectivities with E-I combinations producing oscillations with DFA > 0.6 indicated with black squares. (**h**) For networks close to the critical state, *fE*/*I* correctly assigns their activity to be either inhibition dominated (*blue*), excitation dominated (*red*) or in balance (*white squares*). For strongly inhibition- or excitation-dominated networks, DFA is not significant and the presented method is not applicable (*gray regions*, where DFA $\le$ 0.6). (i) *fE*/*I* is associated strongly with *sE/I* (calculated for networks between *sE/I* = 1–3 that pass the inclusion criterion of DFA > 0.6). (j) *fE*/*I* exhibits stronger correlation with *κ* than with *sE*/*I*.

The observed strong coupling in the CROS model between *sE*/*I*, oscillation amplitude, and LRTC suggests that it could be possible to derive a measure of network-level E/I from oscillatory activity. However, the *sE*/*I* modelling also showed that neither the amplitude nor LRTC alone are sufficient to determine whether a network operates in the inhibition- or excitation-dominated regime (Fig. 2a). Firstly, oscillation amplitude (i.e., the square root of the power) exhibited an approximately linear relationship with *sE*/*I* (Fig. 1e) and, thus, cannot define a reference point for when the E/I structure is balanced. Secondly, the inverse U-shaped relationship between *sE*/*I* and LRTC (Fig. 1g) implies that different *sE*/*I* ratios can lead to the same LRTC. To overcome these limitations, we hypothesize that in spite of a fixed structural connectivity in a network there will be fluctuations in oscillation amplitude and LRTC that relate to the *sE*/*I*. This is plausible because networks exhibiting LRTC, show periods of high and low amplitude (Fig. 1c). Importantly, if these fluctuations in oscillation amplitude and LRTC follow the relationships seen in Fig. 1e,g, then it also follows that a functional form of E/I ratio (*fE*/*I*) can be estimated from the covariance of amplitude and LRTC within a signal and that this *fE*/*I* should be related to *sE*/*I*. This would predict a positive relationship between amplitude and LRTC for sub-critical networks, and a negative relationship for super-critical networks (Fig. 2a). Importantly, while *fE*/*I* is expected to correlate with *sE*/*I*, the structural and functional expressions of E/I balance are not necessarily identical. In analogy, functional connectivity derived from BOLD-signal fluctuations measured with fMRI merely shows moderate correlation with structural connectivity in large-scale brain networks as measured with diffusion tension imaging^29^. In other words, structure affects function but they are not the same.

One method to calculate the covariance of amplitude and LRTC would be to split the signal into segments, calculate these measures for each segment, and then correlate them. However, due to the length of data required for each segment to get a reliable estimate of the DFA exponent this would require recordings longer than 10 minutes^21^. Moreover, it may be that E/I ratio as expressed in activity is changing on time scales of seconds rather than tens of seconds. Thus, to develop a sensitive functional E/I measure, we seek a method that estimates LRTC on short time-scales. To this end, we use the fluctuation function from the detrended fluctuation analysis, *F*(*t*), which is a candidate estimator for the DFA exponent on short-time scales. For scale-free signals, an increase in LRTC will lead to a corresponding increase in the fluctuation function on all time-scales. However, correlating the DFA exponent with the mean fluctuation, <*F*(*t*)>, in a certain window size (Fig. 2b), shows it to be a poor estimator of DFA exponents. Here, both measures are obtained using the entire signal. This is because the fluctuation function is influenced by both the auto-correlations and the amplitude of the signal. This is illustrated in Fig. 2c, where doubling the magnitude of a signal, does not alter the LRTC within the signal, but does double the mean fluctuation for each window size. Therefore, for the fluctuation function to be a good estimator of the DFA exponent, it is necessary to remove the influence of amplitude on the fluctuation function by calculating the normalized fluctuation function *nF*(*t*). The mean normalized fluctuation function (<*nF(t)>*) is a reliable estimator of the DFA exponent of that signal (Fig. 2d) and can be calculated on time-scales as short as a few seconds. *nF*(*t*) does not require calculation on many different time scales in order to estimate DFA exponent as by assuming a linear (scale-free) relationship there is a one-to-one mapping between *nF*(*t*) and DFA exponent (Fig. 2d).

Each analytical step from oscillations to the detrended amplitude-normalized signal profile that is necessary for calculating the *nF*(*t*) is illustrated for one example network in Fig. 2e: The signal is band-pass filtered (*i*), the amplitude envelope extracted (*ii*), the signal profile, *S*, of the amplitude envelope, *A*, is calculated as the cumulative sum of the demeaned amplitude envelope (*iii*):

$$S(t)=\mathop{\sum }\limits_{k=1}^{t}(A(k)-\langle A\rangle )$$

and split into windows of a certain size (e.g., 5 seconds) as in the DFA calculation^21,30^. Importantly, as an additional step (*iv*), each of these signal-profile windows is divided by the mean of the amplitude envelope for that window calculated during step (*ii*). These amplitude-normalized windows are then detrended (*v*) and, subsequently, we calculate the normalized fluctuation function *nF*(*t*) for each window as the standard deviation of the amplitude-normalized signal profile (*vi*). By calculating *nF*(*t*) as a proxy of LRTC on short time scales and amplitude on overlapping windows of 5 seconds, it is possible to test whether the hypothesized relationship between LRTC and amplitude holds for networks with different *sE*/*I* (Fig. 2a). These associations were indeed observed in networks that were representative of inhibition-dominated, balanced or excitation-dominated regimes (Fig. 2f). Thus, we can define a functional excitation-inhibition ratio as:

$$fE/I=1-{r}_{{W}_{amp},{W}_{nF(t)}}$$

Where ${r}_{{W}_{{amp}},{W}_{{nF}(t)}}$ is the Pearson correlation between the set of windowed detrended amplitude-normalized signal profiles, *w*_*nF(t)*_, and the set of windowed amplitude values, *w*_*amp*_. Inhibition-dominated networks will hence be assigned *fE*/*I* below 1, excitation-dominated networks *fE/I* greater than 1, and critical networks will have *fE*/*I* = 1. We use a DFA > 0.6 inclusion criterion for signals before computing the *fE*/*I* because networks without LRTC will not show a co-variation of amplitude and the fluctuation function (see the gray zones in Fig. 2a); however, this should not be a strongly limiting factor of the method given the robustness of LRTC in human resting-state oscillations^31^. Corroborating this observation with the simulated data, we found that the *fE*/*I* correctly assigned networks to be excitation or inhibition dominated (Fig. 2g,h), and that *sE*/*I* and *fE*/*I* were strongly correlated (*r*(60) = 0.83, *p* < 10^−16^, Fig. 2i).

Structural E/I could be defined also in other ways; however, the method and subsequent results reported here are not dependent on the exact definition, e.g., the correlation between *sE*/*I* and *fE*/*I* does not change notably with the exact definition of *sE*/*I* (if *sE*/*I* = (E→E)/(E→I + I→ E + I→I): *r*(60) = 0.82, *p* < 10^−15^. *sE*/*I* = (E→E + I→I)/(E→I + I→E): *r*(60) = 0.82, *p* < 10^−15^). What matters is that for a certain structural level of excitation and inhibition—as indicated by the highlighted squares on the diagonal in Fig. 2g—neuronal network activity exhibits a specific covariance between the level of activity and its temporal dynamics (Fig. 2h), which makes it possible to calculate a functional form of E/I directly from ongoing oscillations in the network activity. Importantly, while our model produces activity with spectra that are similar to eyes-closed rest EEG recordings with a dominant peak in the alpha-frequency band (Fig. 1d), we found that applying the analysis to other classical frequency bands, we obtained similar results (*Pearson* (*sE/I*, *fE/I*) Delta: *r*(84) = 0.81, *p* < 10^−19^; Theta: *r*(55) = 0.80, *p* < 10^−13^; Beta: *r*(53) = 0.81, *p* < 10^−13^; Gamma: *r*(45) = 0.81, *p* < 10^−11^). These results suggest that the method does not require prominent peaks in the power spectrum but merely that the neuronal activity emerges from a network that is near the critical point (as defined by DFA > 0.6).

Equating *sE*/*I* to the ratio of synapses of a certain type provides a straightforward manner for controlling E/I balance in the CROS networks. However, when defined in this way, *sE*/*I* neglects other properties of a neuronal network that can affect activity propagation and which, in turn, could influence *fE*/*I*. For example, networks with similar structural E/I ratio could have topologies that differ in how they facilitate activity propagation and, thus, the functional expression of E/I balance. To investigate this, we related *fE*/*I* directly to a measure of activity propagation that is known to reflect E/I balance, namely neuronal avalanches^23^. Networks with balanced E/I and critical dynamics exhibit a power-law distribution of avalanche sizes (Supplementary Fig. S1a). In sub-critical networks, the strong inhibition prevents large avalanches from occurring and, thus, the avalanche-size distribution is below that of a critical network. In super-critical networks, the excess excitation often prolongs the avalanches, leading to an avalanche-size distribution with values above that of a critical network (Supplementary Fig. S1a). To quantify the proximity of the measured avalanche-size distribution to a power law, we used the *κ* index (Supplementary Fig. S1b, Supplementary Methods)^32^. Critical dynamics of avalanches (*κ* = 1.0) were indeed found for an intermediate value of *sE*/*I* (Supplementary Fig. S1c). However, we found that networks with similar *sE*/*I* exhibit a wide range of *κ* values (Supplementary Fig. S1c), confirming that there are factors not captured in our measure of *sE*/*I* that affect activity propagation in the form of neuronal avalanches. Importantly, when we correlated *fE*/*I* to *κ* we found a much stronger relationship than that of *fE*/*I* with *sE*/*I* (*Pearson* (*κ*, *fE/I*) *r*(60) = 0.96, *p* < 10^−33^, Fig. 2j) showing that *fE*/*I* accurately distinguishes between the inhibition-dominated subcritical regime and the excitation-dominated supercritical regime. Thus, *fE*/*I* is better seen as an index of the balance between excitatory and inhibitory activity that emerges out of the network structure, rather than a direct measure of the E/I ratio of the network structure itself. This suggests that *fE*/*I* could be used to infer the functional E/I ratio from the network dynamics regardless of the mechanisms affecting these dynamics.

### Functional E/I ratio in humans is balanced in the eyes-closed rest state and sensitive to synaptic modulation

**Figure 3:**
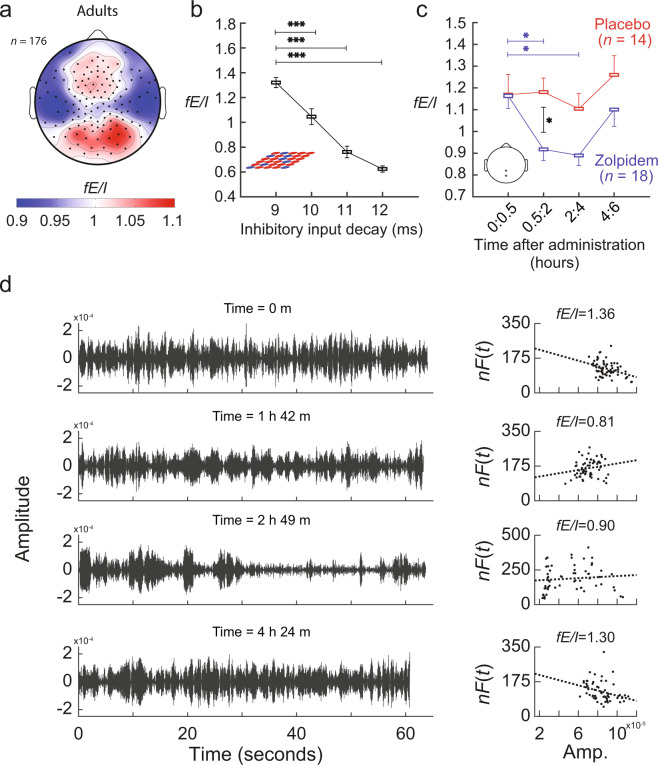
*fE/I* is balanced in healthy populations and can detect pharmacologically induced shifts in excitation/inhibition ratio. (**a**) Grand average *fE/I* topography during eyes-closed rest (*n* = 176). (**b**) Increasing inhibitory decay rate in the model is associated with decreasing *fE/I*. (**c**) Eighteen subjects taking zolpidem, which increases inhibitory input decay constant, show a significant decrease in *fE*/*I* and a return to baseline values after 4–6 hours both relative to baseline (*blue asterisks*) and relative to the placebo control (*black asterisks*). The bipolar Oz-Pz electrode position measured is indicated on the inset. Bonferroni-corrected *P*-values are indicated as * <0.05. (**d**) Filtered signal (left column) and *fE*/*I* (right column) shown for a single subject (zolpidem condition) at four time points.

Our model results suggest that if mass-neuronal activity in the human brain is poised close to the critical state during rest^18,24,33,34^, then *fE*/*I* should be close to one. To corroborate this prediction, we recorded EEG during eyes-closed rest in 176 healthy adults and calculated the *fE*/*I* values in the alpha band. At the group level, the method proved applicable with the DFA-threshold criterion of >0.6 met by 84% of the signals and revealed that *fE*/*I* was highest in frontal, parietal and occipital regions (Fig. 3a). Interestingly, averaging *fE*/*I* across all electrodes to get a “whole-brain estimate” of functional E/I ratio revealed that the operating point of the activity varied across subjects between sub- and super-critical regimes with a mean *fE*/*I* close to one (0.99 ± 0.17, mean ± SD). Together, this indicates that a balanced E/I ratio is indeed typical for whole-brain dynamics at rest in non-clinical populations.

Next, we hypothesized that not only structural connectivity would influence *fE*/*I* (Fig. 2g-i), but also changes to synaptic functioning^9^. To investigate this, we extended the model so that the decay constants of excitatory and inhibitory inputs could be changed independently. Simulations were run for the same networks used in the simulations for Fig. 1, but now using three different inhibitory input decay constants (10, 11, and 12 ms) and compared to that of the baseline networks with 9 ms decay constant (see Methods) to show how an increase in inhibitory input decay alters network dynamics. The *fE*/*I* showed a significant decrease compared to baseline (Fig. 3b) for all decay-time constants (9 ms: 1.35 ± 0.04, mean ± SEM of *fE*/*I* for 42 combinations of excitatory-inhibitory connectivity; 10 ms: 1.05 ± 0.06, *p* < 10^−6^; 11 ms 0.76 ± 0.05, *p* < 10^−7^; 12 ms: 0.61 ± 0.03, *p* < 10^−7^; Wilcoxon signed-rank test, Bonferroni corrected for 3 comparisons). Thus, our measure of *fE*/*I* is sensitive not only to structural but also to synaptic influences on excitation-inhibition balance in the model.

To confirm that our method can detect changes in E/I ratio through pharmacological manipulation, we conducted a challenge in healthy adults comparing placebo (*n* = 14) to zolpidem (*n* = 18, 10 mg orally)—an E/I-modulating agent that enhances GABAergic synaptic inhibition by increasing the decay-time constant of GABA_A_ receptor-mediated postsynaptic currents^35^. In accordance with zolpidem’s pharmaco-kinetic properties^36^, and corroborating the model simulations (Fig. 3b), we observed a significant reduction of *fE*/*I* in zolpidem compared to placebo, between 30 min and 2 h after the administration (bipolar Oz-Pz electrode, Wilcoxon rank-sum test, Bonferroni corrected for 4 comparisons, zolpidem vs Placebo, 0–0.5 h: *p* = 0.8; 0.5–2 h: *p* = 0.004; 2–4 h: *p* = 0.02; 4–6 h: *p* = 0.25) (Fig. 3c). The zolpidem group also showed significant decreases compared to the baseline block (0–30 min), between 30 min and 4 h after the administration (Wilcoxon signed-rank test, mean ± SEM, Bonferroni corrected for 3 comparisons, 0–0.5 hours: 1.16 ± 0.06; 0.5–2 hours: 0.92 ± 0.05, *p* = 0.007; 2–4 hours: 0.89 ± 0.05, *p* = 0.003; 4–6 hours: 1.10 ± 0.08, *p* = 0.62). Note that the high baseline *fE/I* values relate to the Oz-Pz bipolar electrode measured during the pharmacological intervention and this is the scalp region with the highest *fE*/*I* values (cf., Fig. 3a). Looking at a single subject (Fig. 3d), we can see how observable reductions in amplitude and changes in amplitude dynamics after administration of zolpidem are mirrored by changes in the relationship between windowed amplitude and *nF*(*t*). Together, these findings suggest applicability of the *fE*/*I* method for clinical purposes and treatment-effect monitoring.

Shifts in E/I ratio may also occur between brain states under the action of neuromodulatory systems ^37,38^, in a manner analogous to the zolpidem pharmacological manipulation. While the eyes-closed resting state has been associated with critical balance, neuronal dynamics shift to a subcritical state during eyes-open rest ^39,40^, which is associated with a lower E/I ratio^25^. We compared the whole-brain average *fE*/*I* values in the alpha band for the 176 EEG eyes-closed rest recordings with a group of 73 healthy adults that underwent eyes-open rest EEG recordings. We found *fE*/*I* to be decreased during eyes-open rest compared to eyes-closed rest (Wilcoxon rank-sum test, mean ± SEM, eyes-closed rest: 0.99 ± 0.01, eyes-open rest: 0.81 ± 0.02, *p* < 10^−14^, Supplementary Fig. S2). Thus, *fE*/*I* can also track changes in E/I ratio between cortical states.

### ASD is characterized by alterations in critical brain dynamics

**Figure 4:**
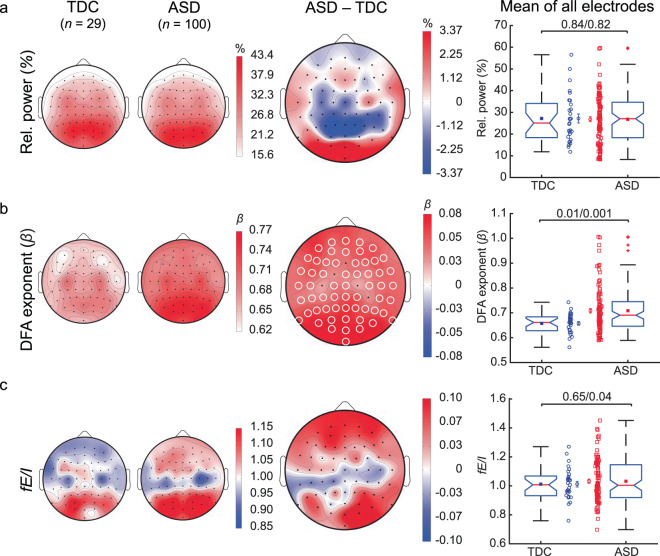
Alpha oscillations in ASD are characterized by strong LRTC and large variability in *fE/I*. Grand-average topographies for the EEG biomarkers are shown for TDC (first column), ASD (second column), and for ASD-minus-TDC (third column). (**a**) Relative power (RP) showed the characteristic occipito-parietal distribution both in TDC and ASD. (**b**) Whole-brain average and variability of LRTC quantified by the DFA exponent were both larger in ASD compared to TDC. (**c**) A pronounced variability of whole-brain *fE*/*I* was evident in ASD. White circles on the topographies represent significant channels (i.e., *p-*value < 0.05, using Wilcoxon rank-sum test and FDR correction). The fourth column shows individual-subject values, boxplots, and mean and SEM for TDC (*blue circles*) and ASD (*red squares*). Comparisons represented in boxplots were based on the average value of the EEG biomarkers across all 64 electrodes, each data point represents one subject (whole-head average; *p*-values are from Wilcoxon rank-sum test (mean)/Levene’s test (variability).

To test the hypothesis that E/I imbalances characterize ASD ^4,41^, we applied the algorithm to eyes-closed rest EEG recordings in non-medicated children with ASD (ASD_all_; *n* = 100, 7–15 years) and age-matched typically developing children (TDC; *n* = 29) (Table 1 and Supplementary Table S1). The children with ASD had lower total IQ (*p* < 0.001) than TDC (Table 1). As with the CROS-model activity, we analyzed relative power (RP), LRTC, and *fE*/*I* in the alpha band of the EEGs. The topography of RP showed the characteristic occipito-parietal distribution of eyes-closed rest recordings both in TDC and ASD without between-group differences in mean or variance of whole-brain averages (Fig. 4a; Wilcoxon rank-sum test, mean ± SEM; *RP*_ASDall_ = 26.8 ± 1.1% and *RP*_TDC_ = 27.2 ± 2.1% *p* = 0.84; Levene’s test of variance: *p* = 0.82). The scalp distribution of LRTC quantified by DFA was also similar between TDC and ASD, but whole-brain average and variability were both larger in ASD (Fig. 4b; *β*_ASDall_ = 0.70 ± .01, and *β*_TDC_ = 0.65 ± 0.01 *p* = 0.01; Levene’s test: *p* = 0.001). The scalp topography of *fE*/*I* of alpha oscillations showed similar distributions in ASD_all_ and TDC. A pronounced variability of *fE*/*I* was evident in ASD_all_ (Fig. 4c; Levene’s test: *p* = 0.04), characterized by more extreme values of *fE*/*I* in both directions away from the balance point although between-group differences of whole-brain average *fE*/*I* were not significant (Fig. 4c; *fE*/*I*_ASDall_ = 1.03 ± 0.02 and *fE*/*I*_TDC_ = 1.01 ± 0.02, *p* = 0.65).

**Table 1:** Clinical characteristics of children with ASD and typically developing children.

	TDC	ASD	*t* (*df*)/W, z	*p-*value
*n*	29	100	—	—
Males/female	14/15	73/27	—	—
Age (mean ± SEM)	10.3 ± 0.28	10.5 ± 0.23	−0.64 (67)	0.524
ADOS (mean ± SEM)	N/A	9.35 ± 0.38	—	—
TIQ (mean ± SEM)	120.6 ± 2.63	101.4 ± 2.08	5.705 (64)	<0.001
SRS-T (mean ± SEM)	43 ± 0.73	75.26 ± 1.02	406, −8.06	<0.001

Mean values and comparison’s statistics (t-test for parametric data (*t*, *df*) and Wilcoxon rank-sum test for non-parametric data (W, z). Demographics and EEG mean biomarker values per subject included in our study can be found on Supplementary Table S1. ADOS (Autism Diagnostic Observation Scale); TIQ (Total Intelligence Quotient); SRS-T (Social Responsiveness Scale, (T-scores). N/A (not applicable).

Albeit IQ may be considered part of the disorder rather than a confounding factor^42^, we performed an ANCOVA test to investigate whether the above findings could be explained by the differences in IQ. This analysis did not change the outcomes on whole-brain averaged RP (*F*(1,124) = 0.28, *p* = 0.59, partial *η*2 =0.002), nor for *fE*/*I* (*F*(1,124) = 0.19, *p* = 0.66, partial *η*2 = 0.002); however, the difference in means for LRTC is merely a trend after adjusting for TIQ scores (*F*(1,124) = 3.2, *p* = 0.07, partial *η*2 = 0.026). Together, our findings suggest that larger variability of LRTC and *fE*/*I* contribute to the physiological heterogeneity of ASD.

### ASD without visual EEG abnormalities show elevated fE/I

Visual EEG abnormalities are common in ASD and have been proposed to indicate aberrant neuronal excitability and influence network-level E/I^5,6,43^. To test this hypothesis, we performed qualitative EEG inspection for abnormalities and investigated their association to relative power, LRTC, and *fE*/*I*. For a comprehensive analysis of visual EEG patterns, we used the EEG classification of Luders & Noachtar^44^. Consistent with previous studies^45,46^, we identified EEG abnormalities in 46% of ASD cases (ASD_abn_, *n* = 46) ranging from slowing of activity (*n* = 38) to epileptiform abnormalities (*n* = 8). Of note, none of the subjects with ASD had suffered from seizures nor had received a diagnosis of epilepsy. No visual abnormalities were found in any of the TDC subjects.

**Figure 5:**
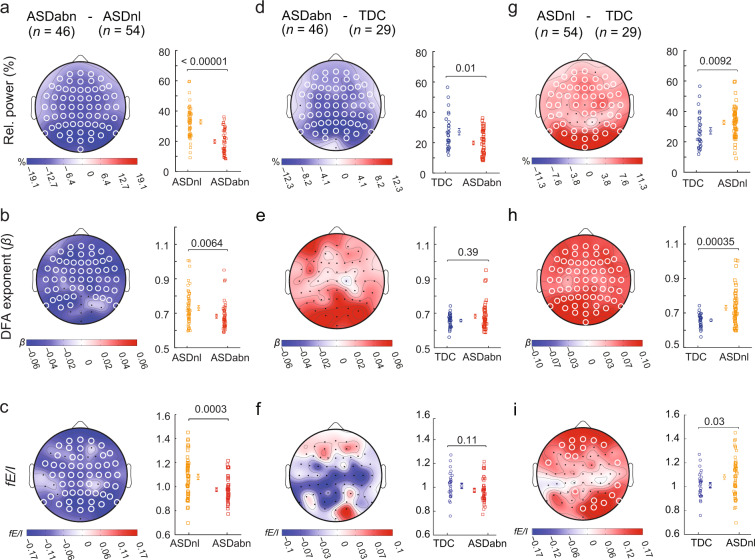
ASD without visual EEG abnormalities show strong LRTC and elevated *fE*/*I*. Compared to the children with ASD and a normal EEG (ASD_nl_), subjects with EEG abnormalities (ASD_abn_) had lower relative power in the alpha band (**a**), weaker LRTC (**b**) and lower *fE*/*I* (**c**). Compared to TDC, ASD_abn_ had lower RP (**d**), but no significant difference in LRTC (**e**) or *fE*/*I* (**f**). In contrast, ASD subjects without any visible EEG abnormalities (ASD_nl_) showed higher RP than TDC (**g**), stronger LRTC (**h**) and higher *fE*/*I* (**i**). Grand-average topographies are shown for the cohort difference of the indicated comparisons (labels on top). The reported *p*-values are based on the Wilcoxon rank-sum test. White circles on the topographies represent significant channels as in Fig. 4. Comparisons at the left of the topographies were based on the average value of the EEG biomarkers across all 64 electrodes, each data point represents one subject (whole-head average, Wilcoxon rank-sum test).

The ASD subsample with an abnormal EEG had markedly different quantitative EEG compared to the ASD with a normal EEG (ASD_nl_; *n* = 54) across the scalp as reflected in lower relative power (Fig. 5a; whole-brain average *RP*_ASDabn_ = 19.8 ± 1.2% vs. *RP*_ASDnl_ = 32.8 ± 1.4%, *p* < 0.00001), weaker LRTC (Fig. 5b; whole-brain average *β*_ASDabn_ = 0.68 ± 0.01 vs. *β*_ASDnl_ = 0.73 ± 0.01, *p* = 0.006), and lower *fE*/*I* (Fig. 5c; whole-brain average *fE/I*_ASDabn_ = 0.97 ± 0.01 vs. *fE*/*I*_ASDnl_ = 1.08 ± 0.02 and, *p* = 0.0003). ASD with abnormalities also differed from TDC in terms of lower relative power (Fig. 5d; *RP*_TDC_ = 27.2 ± 2.1%, *p* = 0.01); however, neither LRTC or *fE*/*I* reached statistical significance (Fig. 5e; *β*_TDC_ = 0.66 ± 0.01, *p* = 0.39; Fig. 5f; *fE*/*I*_TDC_ = 1.01 ± 0.02, *p* = 0.11). Interestingly, in spite of having no visual EEG abnormalities, we found pronounced differences for ASD_nl_ compared to TDC both in terms of higher relative power (Fig. 5g; *RP*_ASDnl_ = 32.8 ± 1.4% vs. *RP*_TDC_ = 27.2 ± 2.1%, *p* = 0.009), stronger LRTC (Fig. 5h; *β*_ASDnl_ = 0.73 ± 0.01 vs. *β*_TDC_ = 0.66 ± 0.01, *p* = 0.00035) and higher *fE*/*I* (Fig. 5i; *fE*/*I*_ASDnl_ = 1.08 ± 0.02 vs. *fE*/*I*_TDC_ = 1.01 ± 0.02, *p* = 0.03). Taken together, our data show that dissecting the ASD cohort into the presence or absence of EEG abnormalities is essential for revealing strong effects both in the classical relative power and in biomarkers rooted in criticality theory (Figs. 4 and 5).

### Subjects with higher SRS scores tend to have higher fE/I

To determine if the level of core clinical symptoms in ASD had an effect on the *fE/I* estimates, we divided the ASD_all_ group into high and low Social Responsiveness Scale (SRS-t) t-scores using a median split and compared the groups. We found that the ASD group with high SRS scores had a trend towards higher *fE/I* than the ones with low SRS (Supplementary Fig. S3, Wilcoxon rank-sum test, mean ± SEM; *fE/I* ASD_SRS_low_ = 1.0 ± 0.018, *fE/I* ASD_SRS_high_ = 1.06 ± 0.022, *p* = 0.08).

## Discussion

We have introduced a measure of functional E/I ratio (*fE*/*I*) from network activity that is sensitive to both changes in synaptic functioning and network connectivity and which is applicable to non-invasive human EEG recordings^3,8^. We validated the method *in silico*, and applied it to human EEG to find that a near-critical and E/I-balanced regime is typical for human eyes-closed resting-state brain activity in the alpha band^19,27,33^. In addition, we showed that pharmacological enhancement of GABA_A_ receptor-mediated inhibition lowered *fE*/*I* as predicted. Finally, we found evidence to support the notion of E/I imbalances in ASD in that the variance in LRTC and *fE*/*I* is higher compared to TDC. Importantly, whereas children with visual abnormalities in the EEG merely had lower relative power compared to the TDC, the children without epileptiform or other visual changes showed elevated values of relative power, LRTC and *fE*/*I*.

In spite of the comprehensive literature on E/I balance^3–6,11–16^, there is no consensus on how to define or measure E/I ratio, neither functionally or structurally. The design of our measure of *fE*/*I* was guided by the theory of critical brain dynamics, which is rooted in the physics concept of critical phase transitions^23,47,48^. This choice was motivated by the extensive evidence that brain activity at many levels of neuronal organization exhibits near-critical dynamics and that this requires balance between excitatory and inhibitory forces^14,23,49,50^. These forces can be structural in terms of neuronal connectivity or functional in terms of the kinetics of synaptic currents^51^, and the *fE*/*I* was indeed able to track changes in either of these parameters. Interestingly, albeit *fE*/*I* correlated strongly with any of our definitions of structural E/I ratio (*r* > 0.8), the correlation with the Kappa measure of neuronal avalanches was even stronger (*r* > 0.94). Thus, *fE*/*I* is better seen as an index of the emergent balance of excitatory and inhibitory activity that is a consequence of the network structure, rather than a direct measure of the E/I ratio of the network structure itself. This suggests that *fE*/*I* could be used to infer the functional E/I ratio from the network dynamics regardless of the mechanisms affecting these dynamics.

Neuronal avalanches offer another method for estimating the E/I based on criticality theory^23^. One advantage our *fE*/*I* measure has over neuronal avalanches is that it allows an estimate at the single-electrode level, whereas neuronal avalanches assign one E/I level to all or to a pre-defined cluster of sensors or regions of interest when applied to multi-channel data^24,34^. Moreover, because the brain is not in one “state” as a homogeneous system but is composed of interacting networks that may each have their own level of criticality at any given time^52^ the applicability of avalanche-based E/I estimation is questionable when dependent on sensors that span multiple brain regions. This is supported by the clear topographical distributions of *fE*/*I* seen both in the adult and the children cohorts (Figs. 3a, 4c). The scaling exponent of broadband 1/f signals has also been proposed to estimate changes in E/I balance^16^. Through computational and electrophysiological evidence they show that increasing inhibition induces steeper power spectra. One drawback of this method is that similar to measures of excitability such as alpha power (Fig. 2a), it does not provide an absolute value that corresponds to balanced E/I, or the critical point, and comparing 1/f slopes of the same area recorded with different modalities (EEG & MEG) suggests that this would be difficult to achieve^53^. This may reduce its usefulness as it does not allow hypotheses of function related to the critical point to be tested (e.g., increased dynamic range, information transmission, information capacity)^32^.

Non-invasive estimates of E/I have also been proposed using magnetic resonance spectroscopy (MRS) and stimulation methods such as paired-pulse transcranial magnetic stimulation (TMS). MRS can measure the levels of excitatory (glutamate) and inhibitory (GABA) neurotransmitters in a region^54,55^; however, it is not known how these concentrations relate to the power or dynamics of oscillations as investigated in our CROS model and in the human EEG data. Previous attempts to relate GABA levels with features of M/EEG have shown inconsistent results^56,57^, and so future studies will be necessary to investigate the relationship of MRS to *fE*/*I*. Paired-pulse TMS has also been used to look at intracortical facilitation and inhibition, i.e., measuring the excitability of an area. Previous studies have shown a relationship between variability of motor-evoked potential response and the variability or amplitude of alpha oscillation amplitude^58,59^, suggesting that there could be a relationship between *fE*/*I* and paired-pulse TMS, which should be investigated.

We restricted the current analysis to alpha-band oscillations but show that *fE*/*I* values in the CROS model were similar across frequency bands, which we explain by the oscillations at all frequencies being generated in the same *in silico* network. In the human brain, however, different circuits and network mechanisms may underlie oscillations at different frequencies and, consequently, the *fE*/*I* could probe E/I balance specific to the microcircuits producing different oscillations. Alpha oscillations are known to become strongly suppressed by eye opening^27^. Indeed, we found lower *fE*/*I* during eyes-open rest compared to eyes-closed rest. This is consistent with previous studies, showing a shift towards subcritical neuronal dynamics in the eyes-open rest condition^39^. In fact, subcritical dynamics as observed during eyes-open rest may be beneficial during tasks^60^, especially for tasks that require focused attention^49,61,62^. Future studies may use *fE*/*I* to further investigate how performance in cognitive tasks relate to critical brain dynamics and E/I regulation^34,63^.

LRTC in critical brain dynamics are expected to reflect network stability and, therefore, LRTC in addition to *fE*/*I* may constitute a physiological biomarker of network dysfunction in ASD^1,48^. The development of the *fE*/*I* measure was needed as quantification of LRTC alone is insufficient to estimate how network E/I balance is altered (Fig. 2a). Using the novel algorithm, we found that both LRTC and *fE*/*I* variability was larger in ASD than in TDC. This seems to confirm experimental studies implicating both decreased, normal, or increased E/I ratios in ASD^3,4,6,8^. The original hypothesis of ASD as a disorder of elevated E/I was predominantly based upon the frequent concurrence of epilepsy and epileptiform changes^45,64^. We found that the ASD cohort without any visible EEG abnormalities displayed elevated LRTC and *fE*/*I* over multiple scalp regions versus the control sample. This finding was further supported by the within ASD sample comparison (ASD_nl_ vs. ASD_abn_) where the group with EEG abnormalities showed lower power, LRTC and *fE*/*I*.

We can thus far only speculate why seemingly inhibition-dominated networks may be associated with different types of EEG abnormalities. For instance, background slowing of activity is considered a sign of neurodevelopmental delay or perinatal insults^44,65,66^. Intermittent slowing can indicate epileptiform changes in specific circumstances (e.g., cortical malformations or temporal lobe epilepsy)^44,66–69^ or an expression of protective mechanisms against epileptogenicity^70^. Whether EEG abnormalities interfere with information processing and whether they merit (anti-epileptic) treatment is debated^46,71–73^. So far, ASD trials have predominantly focused on agents that enforce inhibition or reduce excitation^6^. Indeed, some of these agents have been suggested to be effective in subsets of patients^6,17^ and our proposed method may be tested as stratification biomarkers to increase applicability. A final aspect for future studies is whether the level of clinical impairment is also reflected in the characteristics of these biomarkers. Our findings may point to a relation between more core symptom severity and elevated *fE/I* but investigation in larger samples is first needed.

In conclusion, we propose that parallel quantification of the critical brain dynamics indices of power, LRTC and *fE/I* may provide a novel theory-based framework to advance understanding of physiological heterogeneity of ASD and related disorders.

## Methods

### Computational model to estimate the excitation-inhibition ratio

#### CRitical OScillations (CROS) model

The CROS model is an *in silico* model that was created by Poil *et al*. (2012) with the aim of having a spiking neuronal network that produced oscillatory activity with long-range temporal correlations in the oscillation amplitude. In this paper, we use an extended version of this model with optimized parameters to investigate the dependence of neuronal network activation patterns on structural connectivity and synaptic parameters. CROS models a network of 75% excitatory and 25% inhibitory integrate-and-fire neurons arranged in a 50 × 50 open grid^25^. Networks differ in their two connectivity parameters, ${C}_{E}$ and ${C}_{I}$, which are the percentage of other neurons within a local range (a square with width = 7 neurons centered on the presynaptic neuron) that each excitatory and each inhibitory neuron connects to, respectively. Connectivity parameters were set between 25–100% at 5% intervals, and 20 different networks were created for each combination of excitatory and inhibitory connectivity – ${C}_{E}$ and ${C}_{I}$. For a full description of the model see Supplementary Methods.

#### Detrended fluctuation analysis of long-range temporal correlations

The detrended fluctuation analysis (DFA) was used to analyze the scale-free decay of temporal (auto)correlations in the amplitude modulation of neuronal oscillations, also known as long-range temporal correlations (LRTC)^30^. The DFA exponent, *β*, is the slope of the fluctuation function shown, e.g., in Fig. 1f. DFA exponents in the interval of 0.5 to 1.0 indicate scale-free temporal correlations, whereas an exponent of 0.5 characterizes an uncorrelated signal. The analytical steps to quantify LRTC using DFA have been explained in detail previously ^21,27^ and are briefly explained in Supplementary Methods.

#### Estimation of excitation/inhibition ratio (*fE*/*I*)

For a full explanation of the rationale behind the algorithm to estimate excitation/inhibition ratio, we refer to the Results section because it is a new method. Here, we merely detail the sequence of analytical steps. To test the relationship between the amplitude and LRTC of the amplitude envelope of an oscillatory signal, it is necessary to have a measure of LRTC on short time-scales that is unbiased by the amplitude of the signal. To this end, we introduce an amplitude-normalized fluctuation function, *nF*(*t*), for a signal that is calculated as follows (Fig. 2d): The signal is band-pass filtered (*i*), the amplitude envelope *A* extracted (*ii*), the signal profile, *S* can then be calculated as the cumulative sum of the demeaned amplitude envelope (*iii*):

1 $$S(t)=\mathop{\sum }\limits_{k=1}^{t}(A(k)-\langle A\rangle )$$

and split into windows of a certain size (e.g., 5 seconds) in exactly the same way as during the DFA calculation^21,30^. Importantly, as an additional step (*iv*), each of these signal-profile windows is divided by the mean of the amplitude envelope for that window calculated during step (*ii*). These amplitude-normalized windows are then detrended (*v*) and, subsequently, we calculate the normalized fluctuation function for each window as the standard deviation of the amplitude-normalized signal profile (*vi*). To calculate the functional excitation/inhibition ratio, *fE*/*I*, perform a Pearson correlation between the amplitude and the normalized fluctuation function for the set of windows *W* (*vii*). *fE*/*I* is then defined as:

2 $$fE/I=1-{r}_{{W}_{amp},{W}_{nF(t)}}$$

All of the signal-processing steps (*i*)-(*vii*), and an illustration of how they affect the signal, are shown in Fig. 2e. Sub-critical networks will have an *fE*/*I* < 1, super-critical networks *fE*/*I* > 1, and critical networks will have *fE*/*I* = 1. We used a DFA > 0.6 inclusion criterion for networks or channels before computing the *fE*/*I* because networks without LRTC will not show a co-variation of amplitude and the fluctuation function (Fig. 2a). For our analyses, *fE*/*I* was calculated for windows of 5 seconds with 80% overlap.

### Validation and clinical application of fE/I method

#### Participants and EEG recordings

Ethical approval was obtained from the corresponding medical ethical committees (VU Medical Center, Utrecht Medical Center, or Leiden University Medical Center). The studies were conducted in accordance with the guidelines and regulations approved by the respective ethical committee and in compliance with the provisions of the declaration of Helsinki and Good Clinical Practice. Written informed consent was received from the participants or their legal guardians prior to inclusion in the study.

#### Adult validation sample

249 healthy adults were included, in a study at VU Amsterdam. Exclusion criteria were a history of neurological pathology or current use of medication. 176 subjects (age 19–56 years, M = 24.4 years, SD = 7 years, 107 females) underwent resting-state EEG recordings with eyes closed, while 73 subjects (age 19–47, M = 24 years, SD = 7.8 years, 49 females) had their eyes open. Resting-state EEGs were recorded for 3–5 minutes using the NetAmps300 amplifier (Electrical Geodesics) and 129-channel HydroCel Geodesic sensor nets. The sampling rate was 1000 Hz and the reference electrode was Cz.

#### Adult zolpidem sample

EEGs recorded from forty medically-screened healthy subjects (age 18–65 years, 3 females) were made available from the Centre for Human Drug Research, the Netherlands. Subjects had taken part in a double-blind randomized study, receiving a capsule orally containing 10 mg zolpidem (20 subjects) or placebo (20 subjects). Subsequently, subjects underwent multiple EEG sessions using a bipolar recording at PZ-OZ along with a separate channel for recording eye artifacts. Each EEG session included 64 seconds of eyes-closed rest. 28 subjects (14 zolpidem) were recorded at 0, 0.33, 1, 1.33, 2, 3, 4, 6, 8, 10 and 12 hours after administration of the capsule. 12 subjects (6 zolpidem) were recorded at 0, 0.5, 1, 1.5, 2, 3, 4, 6, 8, 12 hours after administration. Exclusion criteria for this study were: history or clinical evidence of alcoholism or drug abuse within the 3-year period prior to the screening examination, smoking, concomitant treatment with any medication, or any contraindication or known hypersensitivity to zolpidem.

#### Children with ASD and typically developing children (TDC) samples

EEGs and symptom-scale baseline measurements were collected from two ongoing studies at the developmental disorder unit in the UMC Utrecht (for details see Supplementary Methods). Inclusion criteria for ASD and TDC samples were an age between 7–16 years, IQ > 55, and ability to comply with study procedures. ASD diagnosis required an expert diagnosis according to the Diagnostic and structural manual of mental disorders (DSM) IV-TR^74^ or 5^75^ supported by a clinical score on the Autism Diagnostic Observation Schedule 2 (ADOS-2 module 3 or 4, score ≥ 7) or a subclinical score on the Social Responsivity Scale (SRS-t-score ≥ 60)^76^. An abbreviated form of the Wechsler intelligence scale for children (WISC)-III was used for IQ estimation. Details regarding the clinical scales are provided in Supplementary Methods. Exclusion criteria for the ASD sample were use of psychoactive medication and presence of other major neurological disease such as previous or current epilepsy diagnoses. The TDC sample was recruited from local residents attending non-special education, where a history of behavioral or learning problems, a diagnosis of any neurodevelopmental condition or any other major health issue was an exclusion criterion.

We recruited a total of 126 subjects that met the inclusion criteria for ASD and 37 TDC. From these, 26 children with ASD and 8 TDC were excluded because less than 50% of their recordings were free of transient artifacts or no eyes-closed rest EEG had been recorded. Thus, the final included sample consisted of 129 subjects (42 females): 100 with ASD (7–16 years, M = 10.5 years, SD = 2.3 years, 27 females) and 29 age-matched typically developing children (TDC) (7.4–14.4 years, M = 10.3, SD = 1.5 years, 15 females). EEGs were recorded during 3–5 minutes of eyes-closed rest at the UMC Utrecht using A 64-channel BioSemi EEG system at a sampling rate of 2048 Hz and common mode sense (CMS) reference electrode.

#### Pre-processing and analysis of EEG data

EEG analyses were done using the Neurophysiological Biomarker Toolbox (NBT) (http://www.nbtwiki.net/). All recordings were manually cleaned for artifacts, i.e., noisy channels were discarded and noisy intervals removed. Final recordings’ length is provided in Supplementary Methods. Subsequently, the data were re-referenced to the average reference.

For all samples, spectral power was computed using the Welch method with an 8192-point Blackman window and a frequency resolution of 0.12 Hz. Relative power was calculated by dividing the absolute power in the alpha band with the integrated power in the range 1–45 Hz. The alpha band (8–13 Hz) amplitude envelope was extracted for all analyses. The DFA exponent was fit between 2 and 30 seconds for all datasets except for the zolpidem sample (see below). Finally, the *fE*/*I* was calculated as described above (see ‘estimation for excitation/inhibition ratio) for windows of 5 seconds with 80% overlap.

Specifically, for the adult zolpidem dataset, recordings were down-sampled from 64768 Hz to 1024 Hz and filtered 0.5–70 Hz (4^th^-order Butterworth filter). The DFA exponent was fit between 2 and 10 seconds due to the short length of the signals. The *fE/I* was averaged for all recordings with a DFA exponent >0.6 (87% of all recordings) into four time blocks (0–0.5, 0.5–2, 2–4, 4–6 hours). Only subjects who had at least one recording with DFA exponent >0.6 in each of the four time blocks were kept for the analysis, which resulted in the inclusion of 18 of the 20 subjects measured in the zolpidem condition, and 14 of the 20 subjects measured in the placebo condition.

#### Classification of EEG abnormalities

Additionally, the recordings of the children with ASD and TDC were visually inspected in windows of 10 seconds by a neurologist with training in clinical EEG (neurophysiology and epilepsy) (EJM) and scored according to Luders & Noachtar’s classification of EEG abnormalities^44^. When no abnormalities were detected, the EEG was classified as normal.

#### Statistical analysis

Results are expressed as mean ± SEM. Wilcoxon signed-rank test was used to compare the adult zolpidem data at different time points (repeated measures) based on the *fE*/*I* value of the Pz-Oz electrode (Fig. 3c). For the children samples (ASD and TDC), clinical and demographic comparisons were calculated using a t-test (if normality assumptions were met) or Wilcoxon rank-sum test. Wilcoxon rank-sum test was also used to compare EEG biomarker values between children with ASD and TDC and within the different ASD EEG subgroups (independent measures). Additionally, an ANCOVA test was performed to control for IQ as a covariate. In order to show significance also at the electrode level (64 channels) (*white circles* on topographic plots in Figs. 4 and 5), we used False Discovery Rate (FDR) to correct for multiple testing. For the number of channels in our EEG recordings and to show the widespread scalp distribution of the effects in our data, we set *q* = 0.15 in the FDR corrections (for details regarding the determination of *q* see Supplementary Methods). Importantly, because of the broad scalp-distribution of the effects, we also report comparisons based on the average value of the EEG biomarkers across all 64 electrodes (whole-brain average) and show the individual-subject values in Figs. 4 and 5. For these tests, we averaged the value of EEG biomarkers across all electrodes (thus, there is no multiple-comparison issue). The significance level was set at *p* < 0.05. Finally, to determine if the level of core clinical symptoms in ASD had an effect on the *fE/I* estimates, we used a median split to divide the ASD_all_ group into high and low Social Responsiveness Scale (SRS-t) t-scores and compared the groups performing the Wilcoxon rank-sum test. The significance level was set at *p* < 0.05.

## Supplementary information

Supplementary information.

## Data Availability

Due to privacy regulations of human subjects, we cannot provide the EEG files of the subjects included in our study. However, we provide the individual demographics and EEG mean biomarker values in Supplementary Table S1. Analysis scripts to reproduce the figures and statistics are available at https://figshare.com/projects/Measurement_of_excitation-inhibition_ratio_in_autism_spectrum_disorder_using_critical_brain_dynamics/80408.

## Code availability

The code for running the simulations of the CROS model is available at https://figshare.com/projects/Measurement_of_excitation-inhibition_ratio_in_autism_spectrum_disorder_using_critical_brain_dynamics/80408. The code for the *fE*/*I* algorithm will be publicly available at the time of publication at https://github.com/rhardstone/fEI.
