# Supplementary material for: Measurement of excitation-inhibition ratio in autism spectrum disorder using critical brain dynamics
Source: Sci Rep. 2020 Jun 8;10:9195. doi: 10.1038/s41598-020-65500-4 (PMC7280527; doi:10.1038/s41598-020-65500-4)
Supplement: Supplementary file 1 — Supplementary information. [file 41598_2020_65500_MOESM1_ESM.pdf]

Supplementary Information

Title:

Measurement of excitation-inhibition ratio in autism spectrum disorder using critical brain dynamics

Authors:
Hilgo Bruining[1,2†], Richard Hardstone[3,4†], Erika L. Juarez-Martinez[2,3†], Jan Sprengers[2†], Arthur-Ervin Avramiea[3], Sonja Simpraga[3,5], Simon J. Houtman[3], Simon-Shlomo Poil[5], Eva Dallares[3], Satu Palva[6], Bob Oranje[2], J. Matias Palva[6,7], Huibert D. Mansvelder[3], Klaus Linkenkaer-Hansen[3*].

* Corresponding author: Klaus Linkenkaer-Hansen. Department of Integrative Neurophysiology, Center for Neurogenomics and Cognitive Research, (CNCR), VU University Amsterdam, 1081 HV Amsterdam, Netherlands. Tel +31 (0) 20 59 86479. klaus.linkenkaer@cncr.vu.nl
† Shared author position

Supplementary Methods

Computational model to estimate the excitation-inhibition ratio

CRitical OScillations (CROS) model

The CROS model was introduced by Poil et al. (2012)[25] and in this paper we use an extended version of this model with optimized parameters. Here we describe the full model, and highlight any extensions and parameter changes that have been made. CROS models a network of 75% excitatory and 25% inhibitory integrate-and-fire neurons arranged in a 50x50 open grid[25]. Networks differ in their two connectivity parameters, C_E and C_I, which are the percentage of other neurons within a local range (a square with width = 7 neurons centered on the presynaptic neuron) that each excitatory and each inhibitory neuron connects to, respectively. Connectivity parameters were set between 25–100% at 5% intervals, and 20 different networks were created for each combination of C_E and C_I. Border neurons had fewer connections because these neurons had a lower number of neurons in their local range. Within the local range, connection probability decreases exponentially with distance. More specifically, the probability, P, of a connection at a distance r was given by:

$$P(r) = \min(\alpha e^{-r}, 1)\,, (1)$$

where α is a constant whose value is optimized separately for excitatory and inhibitory neurons such that the overall connectivity probability within a neuron's local range is equal to C_E or C_I, depending on whether the neuron is excitatory or inhibitory. For example, in the case of an excitatory neuron i, with connectivity probability C_E, where the set of neighboring neurons within

the local range is J, and $|J|$ is the number of neighbors, α will have to satisfy the following equation:

$$\sum_{j\in J} P(r_j) = \sum_{j\in J} \min(\alpha e^{-r_j}, 1) = C_E|J|, (2)$$

As such, we use the Nelder-Mead optimization algorithm [62] to determine the value of α which minimizes the following function:

$$f(\alpha) = \left|\sum_{j\in J} \min(\alpha e^{-r_j}, 1) - C_E|J|\right|, (3)$$

Structural E/I

As connections are determined probabilistically, small differences in the number of synapses can occur for networks with the same connectivity parameters. To account for this, we defined the structural E/I (*sE/I*) of a network to be the ratio between the number of synapses in a network that enhance transmission of activity throughout the network (E->E), and the number of synapses that inhibit transmission (I->E).

Neuron model

Neurons were modeled using a synaptic model integrating received spikes, and a probabilistic spiking model. Each time step (*dt*) of 1 ms starts with each neuron, *i*, updating the excitatory ($I_{i_{exc}}$) and inhibitory ($I_{i_{inh}}$) inputs with spikes received from excitatory ($J_{i_{exc}}$) and inhibitory ($J_{i_{inh}}$) presynaptic neurons respectively, together with an exponential synaptic decay:

$$I_{i_{exc}}(t + dt) = \left(I_{i_{exc}}(t) + \sum_j^{J_{i_{exc}}} W_{ij} S_j\,(\mathrm{t})\right)\left(1 - \frac{dt}{\tau_{I_{exc}}}\right), (4)$$

$$I_{i_{inh}}(t + dt) = \left(I_{i_{inh}}(t) + \sum_j^{J_{i_{inh}}} W_{ij} S_j\,(\mathrm{t})\right)\left(1 - \frac{dt}{\tau_{I_{inh}}}\right), (5)$$

Where the weights W_{ij} are fixed, depending on the type of the pre- and post-synaptic neuron, $\tau_{I_{exc}}$ and $\tau_{I_{inh}}$ represent the decay constants of excitatory and inhibitory inputs respectively, and S is a binary vector, with $S_j = 1$ if the pre-synaptic neuron j fired in the previous time step, and $S_j = 0$ otherwise.

The activation of a neuron A_i, is then updated with these excitatory and inhibitory inputs, together with an exponential decay, τ_P, and a baseline activation level A_0:

$$A_i(t + dt) = (A_i(t) + I_{i_{exc}}(t) + I_{i_{inh}}(t))\left(1 - \frac{dt}{\tau_P}\right) + A_0 \frac{dt}{\tau_P}, (6)$$

The spiking probability P^s is calculated as a function of the neuron activation A_i at the current time step, as follows:

$$P_i^s(t) = \begin{cases} 0, & A_i(t) < 0 \\ A_i(t), & 0 \le A_i(t) \le 1, \\ 1, & A_i(t) > 1 \end{cases} \quad (7)$$

We determine whether the neuron spikes with the probability P_i^s. If a neuron spikes, the neuron activation A_i is reset to the reset value, A_r. At the next time step, all neurons that it connects to will have their input updated again according to Equations 4 and 5.

Model parameters

All of the parameters for the model were the same as the original paper [25] apart from the synaptic weights, for reasons described below. Neuron model: ($\tau_{I_{exc}} = \tau_{I_{inh}}$ = 9 ms), Synaptic model: Excitatory neurons (τ_P = 6 ms, A_0 = 0.000001, A_r = -2), Inhibitory neurons (τ_P = 12 ms, A_0 = 0, A_r = -20).

Model parameters were not constrained by how biologically realistic they were, but instead were tuned in order to improve the range and stability of the long-range temporal correlations. To tune the synaptic weights from the original model, an evolutionary algorithm [63] was applied. The parameters that could vary were the 2 connectivity parameters, C_E and C_I (taking values between 0–100%) and the natural logarithm of the magnitude of the 4 synaptic weights, W_{EE}, W_{IE}, W_{EI}, and W_{II} (taking values between -5 and 1). For each run, a fitness value was calculated based on the avalanches size (κ_{size}) and duration distributions ($\kappa_{duration}$) and the LRTC (see 'Neuronal avalanches' and 'Detrended fluctuation analysis of long-range temporal correlations' below).

$$fitness = \frac{1}{|1-DFA|+|1-\kappa_{size}|+|1-\kappa_{duration}|} \quad (8)$$

The optimum weights (W_{ij}, connecting the presynaptic neuron *j* to the postsynaptic neuron *i*) found by the algorithm were (W_{EE} = 0.0085, W_{IE} = 0.0085, W_{EI} = -0.569, W_{II} = -2).

Modelling the effect of zolpidem

We maintained $\tau_{I_{exc}}$ at 9 ms, whereas the decay constant of inhibitory inputs $\tau_{I_{inh}}$ was set to increasing levels from 9 ms (baseline) to 10 ms, 11 ms, 12 ms. For each value of $\tau_{I_{inh}}$, we ran all combinations of excitatory/inhibitory connectivity, ranging between 25% and 100% at a 5% spacing, with 20 networks per parameter combination, for a duration of 1000 seconds, at a simulation time step of 1 ms. Only combinations of excitatory/inhibitory connectivity with network average DFA > 0.6 at all levels of $\tau_{I_{inh}}$ were used to assess the effect of $\tau_{I_{inh}}$ on *fE/I.*

Neuronal avalanches

To get a measure of the activity dynamics of the neuronal network, we applied neuronal avalanche analysis. A neuronal avalanche was defined as a period where neurons are spiking, surrounded by time-steps where no neurons are spiking. The size of the avalanche is the number of spikes during this period, and the avalanche duration is the duration of the period. We visually inspected the

avalanche size and duration distributions and applied both least-square fitting, as well as maximum likelihood method fitting to those showing log–log linear scaling[64]. This preliminary analysis pointed to power-law exponents of -1.5 for size and -2.0 for duration, in agreement with previous literature on neuronal avalanches[23,65]. However, our model clearly did not produce log–log linear scaling for all parameter combinations and, therefore, we could not use maximum likelihood fitting and power-law exponents to identify subcritical or supercritical dynamics. Instead, we quantified the similarity to a reference power-law distribution using the κ index[32]. This calculates the difference between the distribution of our data and a power-law by calculating the average difference of the cumulative distribution of a power-law function, *P*, (with exponent -1.5 for size and -2.0 for duration) and that of our experimental data, *A*, at 10 equally spaced points on a logarithmic axis (λ) and adding one.

$$\kappa = \frac{1}{10}\sum_{i=1}^{10}(P(\lambda_i) - A(\lambda_i)) + 1 \qquad (9)$$

A subcritical distribution is characterized by $\kappa < 1$, and supercritical distribution by $\kappa > 1$, whereas $\kappa = 1$ indicates a critical network.

Detrended fluctuation analysis of long-range temporal correlations

The detrended fluctuation analysis (DFA) was used to analyze the scale-free decay of temporal (auto)correlations in the amplitude modulation of neuronal oscillations, also known as long-range temporal correlations (LRTC)[30]. The DFA exponent, β, is the slope of the fluctuation function shown, e.g., in Figure 1F. DFA exponents in the interval of 0.5 to 1.0 indicate scale-free temporal correlations, whereas an exponent of 0.5 characterizes an uncorrelated signal. The analytical steps to quantify LRTC using DFA have been explained in detail previously [21,27]. In brief, we first band-pass filter the signal in the band of interest and extract the amplitude envelope by taking the absolute of the Hilbert transform. The signal profile, *S*, of the amplitude envelope, *A*, can then be calculated as the cumulative sum of the demeaned amplitude envelope:

$$S(t) = \sum_{k=1}^{t}(A(k) - \langle A \rangle) \qquad (10)$$

The signal profile is split into overlapping windows of a certain length, *n*. For each window, the fluctuation function, *F*, can be calculated by linearly detrending the window and taking its standard deviation, σ:

$$F(window) = \sigma(detrended\ window) \qquad (11)$$

The mean of the fluctuation function is taken for each window size, $\langle F(n) \rangle$, and plotted for each window size on logarithmic axis. The DFA exponent, β, is the slope of the fluctuation function (Figure 1F). For the optimization of model parameters, the DFA exponent was fit between 5 and 100 seconds.

Measurement of excitation-inhibition ratio in autism spectrum disorder using critical brain dynamics

Children with ASD and typically developing children (TDC): cohort and clinical symptom scales description

The developmental disorder unit in the UMC Utrecht runs a research program for EEG-assisted clinical care. EEG and symptom-scale baseline measurements were collected from two ongoing studies with identical EEG measurement protocols (SPACE (Sensory information Processing in Autism and Childhood Epilepsy) and BAMBI (Bumetanide in Autism Medication and Biomarker, Eudra-CT 2014-001560-35)). ASD diagnosis required an expert diagnosis according to the Diagnostic and structural manual of mental disorders (DSM) IV-TR[59] or 5 [60] supported by a clinical score on the Autism Diagnostic Observation Schedule 2 (ADOS-2 module 3 or 4, score ≥ 7) or a subclinical score on the Social Responsiveness Scale (SRS-t-score ≥ 60)[61].The Autism Diagnostic Observation Schedule – 2 (ADOS-2)[66] is a semi-structured, interactive schedule, performed and scored by trained clinicians. Here, ADOS-2 modules 3 and 4 were used as required for the age range and verbal fluency of our sample. The Social Responsiveness Scale (SRS) is a dimensional parent reported questionnaire that assesses social abilities in daily life of children between 4 and 18 years of age and identifies the presence and severity of social impairment associated with ASD. An abbreviated form of the Wechsler intelligence scale for children (WISC)-III was used for IQ estimation (consisting of subtasks: similarities, vocabulary, block design and figure assembly), if the IQ test had not been administered within the previous 2 years. SRS questionnaires were newly administered in ASD and TDC samples. Previous ADOS and IQ measures were accepted when collected 3 or 2 years before the EEG measurements respectively and were newly administered when older or not present.

Pre-processing and analysis of EEG data

EEG analyses were done using the Neurophysiological Biomarker Toolbox (NBT) (http://www.nbtwiki.net/). The NBT is an open-source MATLAB toolbox that supplies low-level functions for the preprocessing of EEG signals as well as functions for the computation, visualization, and statistical analysis of EEG biomarkers [21]. Additional custom-made scripts developed for this study were implemented in NBT. All recordings were manually cleaned for artifacts, i.e., noisy channels were discarded and noisy intervals removed. Subsequently, the data were re-referenced to the average reference. After the cleaning procedure of the adult validation sample, the 3-minute EEG recordings (n = 25) were reduced to a length between 126 and 177 seconds, with an average of 168 seconds. The 5-minutes recordings (n = 232) were reduced to a length between 180 and 309 seconds, with an average of 280 seconds. For the adult zolpidem sample, the 64-second recordings (n = 40) were reduced to a length between 42 and 64 seconds, with an average of 63 seconds. Finally, an average of 217 seconds (range 61–308 s) per recording were available for analysis for the children with ASD and TDC samples.

Classification of EEG abnormalities

Additionally, the recordings of the children with ASD and TDC were visually inspected in windows of 10 seconds by a neurologist with training in clinical EEG (neurophysiology and epilepsy) (EJM) and scored according to Luders & Noachtar's classification of EEG abnormalities [40]. The EEGs where classified as being abnormal in any of the three levels of abnormalities

described in the classification were present, i.e., grade I: presence of intermittent generalized slowing or only background slowing of activity (background activity in children is age dependent and therefore in our study slowing of activity was considered accordingly); grade II: localized (or lateralized) slowing of activity (frontal, temporal or occipital intermittent rhythmic delta activity are also included in this category), and grade III: presence of epileptiform abnormalities (e.g., spikes, sharp waves, spike and wave complexes, polyspikes and seizures). For a detailed description and examples of the classification and grading system of EEG abnormalities see Luders and Noachtar, 2000. When no abnormalities were detected, the EEG was classified as normal.

Statistical analysis

Results are expressed as mean ± SEM. Wilcoxon signed-rank test was used to compare the adult zolpidem data at different time points (repeated measures) based on the E/I biomarker value between PZ-OZ electrodes (Figure 3C). For the children samples (ASD and TDC), clinical and demographic comparisons were calculated using a t-test or Wilcoxon rank-sum test depending on whether distribution's normality assumptions were met. Wilcoxon rank-sum test was also used to compare EEG biomarker values between children with ASD and TDC and within the different ASD EEG subgroups (independent measures). In order to show significance also at the electrode level (64 channels) (*white circles* on topographic plots in Figures 4 and 5), we used False Discovery Rate (FDR) to correct for multiple testing. To determine the q level of the FDR, we calculated corresponding numbers of channels, which would be false discoveries for different values of q. A $q = 0.05$ would allow no false discoveries if less than 20 out of the 64 channels came out significant from the Wilcoxon rank-sum test (if 20 channels came out as significant, then $q = 0.05$ would imply exactly 1 channel being a false discovery), which we considered too stringent. For $q = 0.15$, having 20 out of 64 channels reach $p < 0.05$ translates into 3 channels being false discoveries. Thus, for the number of channels in our EEG recordings and to show the widespread scalp distribution of the effects in our data, we set $q = 0.15$ in the FDR corrections. Importantly, because of the broad scalp-distribution of the effects, we also report comparisons based on the average value of the EEG biomarkers across all 64 electrodes (whole-brain average) and show the individual-subject values in Figures 4 and 5. For these statistical tests, we averaged the value of EEG biomarkers across all electrodes (and, therefore, there is no multiple-comparison issue). The significance level was set at $p < 0.05$.

Supplementary Figures and Tables

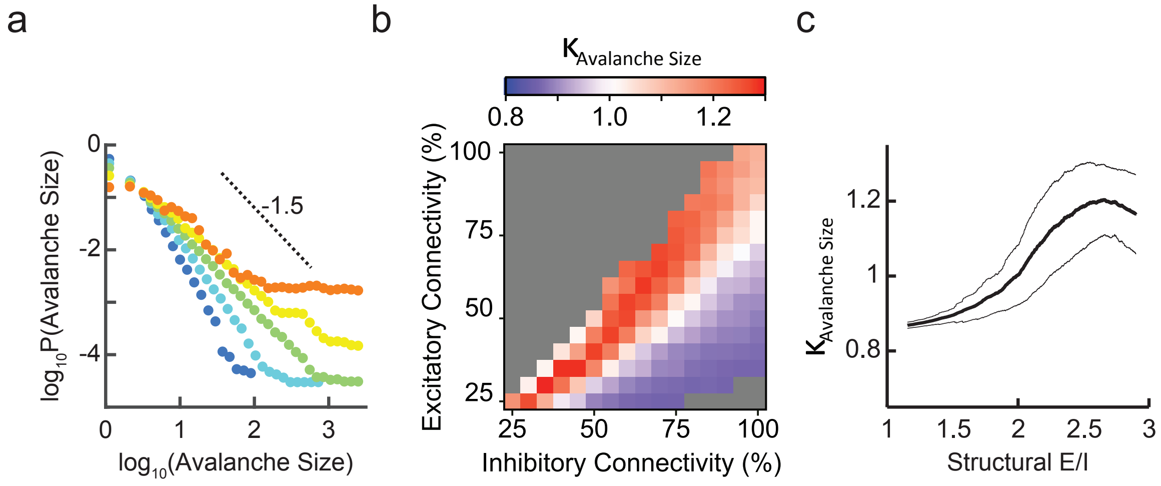

Supplementary Figure S1. Avalanche-size scaling strongly depends on structural E/I.
(a) A neuronal avalanche is defined as contiguous network activity occurring between periods of network silence, and the size of an avalanche is the total number of spikes occurring during the avalanche. The distribution of avalanche sizes changes with the structural E/I for the 5 example networks shown in Figure 1. Specifically, networks with a relatively large number of excitatory connections exhibit both a higher count and a larger size of avalanches. In the balanced, critical state (green), the avalanche-size distribution follows a power law with an exponent of -1.5 (black dashed line). (b) To determine what is the activity-propagation regime in a network, we used the κ index, which compares the actual avalanche distribution with the reference power law, and takes values of $\kappa < 1$ for subcritical networks, $\kappa > 1$ for supercritical networks, and $\kappa = 1$ for critical networks. Computing κ for all connectivity parameter combinations, where sE/I is between 1 and 3, shows that activity propagation depends on a balance between excitation and inhibition across the entire phase space. (c) Specifically, κ increases with sE/I. However, networks with similar sE/I show a broad range of values for κ, suggesting that there are factors other than the ratio of excitatory and inhibitory synapses that contribute to activity propagation.

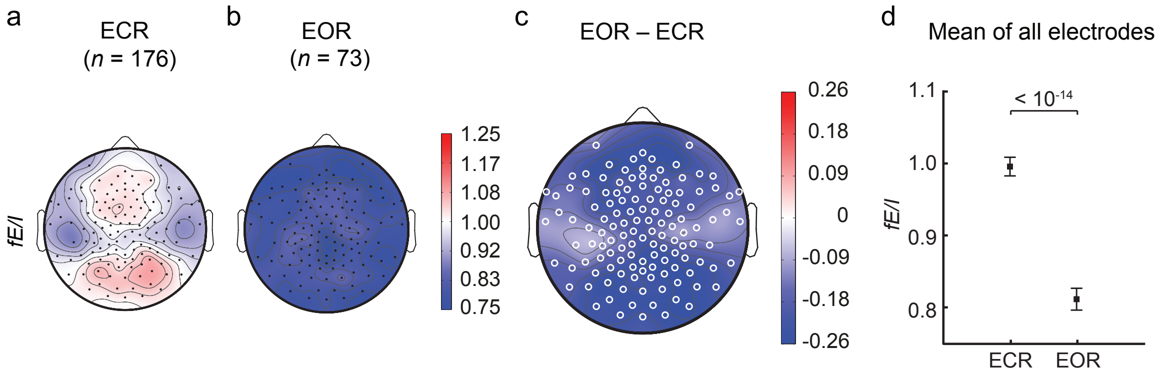

Supplementary Figure S2. *fE/I* can detect changes in E/I ratio between cortical states. Grand average *fE/I* topography during (a) eyes-closed rest (ECR, n = 176) and (b) eyes-open rest (EOR, n = 73). (c) Grand-average topography shown for the difference between eyes-open and eyes-closed rest. White circles represent significant channels as in Figures 4 and 5. The decrease in *fE/I* during eyes-open rest, when compared to eyes-closed rest, is significant across all channels. (d) The comparison between ECR and EOR was based on the whole-head average *fE/I* of all subjects (Wilcoxon rank-sum test, mean ± SEM).

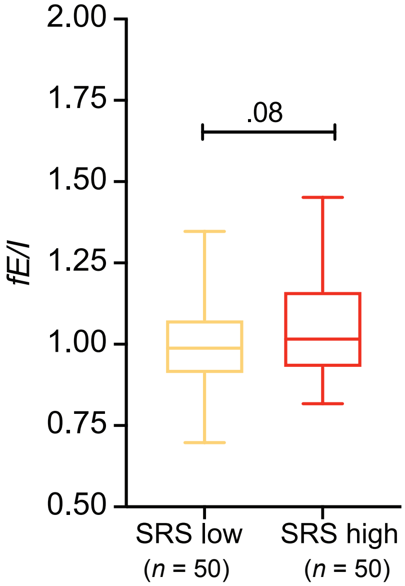

Supplementary Figure S3. The ASD group with high SRS scores tend to have higher *fE*/*I* than the one with low SRS scores. The ASD_{all} group was divided into high and low SRS-t scores using a median split. The means of the whole-head average *fE*/*I* for each group were compared (Wilcoxon rank-sum test).

ID	Group	Age	Gender	EEG score	EI	DFA	Rel. Power
10003	ASD	8.8	M	Abn	0.90	0.69	19.20
10009	ASD	12.6	M	Abn	0.98	0.67	36.20
10015	ASD	10.9	M	Abn	0.98	0.66	13.00
10019	ASD	12.9	M	Abn	0.89	0.70	34.67
10024	ASD	11.8	M	Abn	0.97	0.66	20.09
10026	ASD	12.0	M	Abn	0.88	0.95	26.93
10028	ASD	10.2	M	Nl	1.00	0.78	34.23
10044	ASD	9.2	M	Abn	1.06	0.71	27.93
10057	ASD	10.5	M	Abn	0.97	0.67	22.54
10060	ASD	9.2	M	Abn	0.99	0.62	25.32
10064	ASD	9.6	F	Abn	0.94	0.61	14.79
10069	ASD	9.8	M	Nl	1.45	0.86	42.37
10073	ASD	7.3	M	Nl	1.20	0.83	39.16
10084	ASD	10.3	M	Nl	1.04	0.67	43.33
10086	ASD	12.3	M	Abn	0.85	0.64	22.30
10093	ASD	7.8	M	Abn	0.90	0.72	20.03
10115	ASD	7.6	M	Abn	1.00	0.64	12.02
10126	ASD	12.4	F	Nl	1.12	0.64	38.96
10134	ASD	9.4	M	Nl	1.06	0.69	27.20
10138	ASD	11.1	F	Abn	0.90	0.59	8.68
10149	ASD	10.6	F	Abn	0.93	0.63	16.48
10150	ASD	13.61	M	Nl	1.09	0.79	41.46
10151	ASD	10.0	F	Nl	0.83	0.70	18.15
10161	ASD	7.7	M	Nl	1.18	0.71	23.34
10162	ASD	7.7	F	Abn	1.16	0.67	31.06
10170	ASD	8.2	F	Abn	1.16	0.73	29.54
10175	ASD	11.1	M	Abn	0.89	0.72	21.78
1002	ASD	14.0	F	Nl	1.25	0.69	59.31
1003	ASD	13.9	M	Nl	1.39	0.97	59.70
1004	ASD	7.5	M	Abn	1.02	0.64	28.36
1005	ASD	10.3	M	Abn	1.08	0.68	9.45
1007	ASD	11.6	M	Nl	1.20	0.61	38.34
1009	ASD	13.8	M	Nl	0.95	0.60	37.76
1012	ASD	14.1	M	Nl	1.39	0.77	42.96
1013	ASD	10.0	M	Nl	1.01	0.68	22.87
1015	ASD	8.6	M	Nl	0.70	1.01	24.00
1016	ASD	9.1	M	Abn	1.16	0.74	31.31
1017	ASD	12.3	F	Nl	0.90	0.72	33.12
1018	ASD	10.6	F	Abn	0.91	0.61	12.75
1019	ASD	9.5	M	Nl	1.17	0.72	33.22
1020	ASD	12.4	F	Nl	1.14	0.70	37.61
1021	ASD	7.9	M	Nl	1.35	0.75	39.24
1022	ASD	12.6	M	Nl	1.11	0.65	41.49
1023	ASD	7.8	M	Nl	1.30	0.76	41.24
1025	ASD	11.9	M	Nl	1.33	0.74	27.71
1027	ASD	8.8	M	Abn	1.09	0.66	27.38
1028	ASD	7.4	M	Nl	1.02	0.63	28.13
1029	ASD	8.7	M	Nl	1.01	0.87	32.59

ID	Group	Age	Gender	EEG score	EI	DFA	Rel. Power
1032	ASD	10.0	M	Nl	1.17	0.71	52.11
1033	ASD	12.7	M	Abn	0.93	0.59	8.33
1034	ASD	14.0	M	Abn	0.98	0.89	29.04
1036	ASD	10.6	F	Abn	0.98	0.73	20.16
1037	ASD	12.9	M	Nl	0.94	0.83	24.44
1038	ASD	15.7	M	Nl	0.96	1.00	30.11
1039	ASD	7.7	M	Abn	1.07	0.65	18.54
1040	ASD	7.6	F	Abn	0.94	0.67	15.49
1043	ASD	14.9	M	Nl	0.82	0.67	23.75
1045	ASD	11.0	F	Nl	0.94	0.61	25.00
1046	ASD	10.6	F	Nl	1.15	0.76	34.64
1047	ASD	8.1	F	Abn	0.98	0.62	10.27
1049	ASD	7.1	M	Nl	0.89	0.70	12.41
1050	ASD	8.0	F	Abn	0.82	0.61	8.47
1052	ASD	10.1	F	Nl	1.02	0.66	24.72
1054	ASD	10.4	M	Nl	1.09	0.70	36.74
1056	ASD	8.9	M	Nl	1.18	0.82	32.46
1059	ASD	9.6	M	Abn	1.19	0.78	32.48
1060	ASD	9.8	M	Abn	0.92	0.72	14.79
1061	ASD	10.0	M	Abn	0.92	0.66	15.24
1062	ASD	7.1	M	Nl	1.08	0.60	23.42
1064	ASD	16.0	F	Abn	0.84	0.70	13.39
1066	ASD	11.1	M	Nl	1.14	0.81	36.42
1082	ASD	9.2	M	Abn	0.91	0.76	9.14
1083	ASD	8.2	M	Nl	0.88	0.89	22.16
1084	ASD	8.1	M	Nl	0.94	0.67	31.62
1085	ASD	12.3	F	Nl	0.89	0.60	15.85
1086	ASD	7.0	F	Abn	0.94	0.65	15.40
1087	ASD	8.0	F	Abn	0.89	0.69	11.00
1088	ASD	9.4	M	Abn	1.00	0.67	22.69
1089	ASD	9.1	M	Nl	1.20	0.80	38.18
1091	ASD	12.0	M	Nl	1.08	0.62	38.62
1093	ASD	11.2	F	Nl	0.89	0.61	9.06
1094	ASD	10.4	M	Nl	1.22	0.62	36.41
1095	ASD	11.8	M	Nl	0.92	0.80	28.56
1098	ASD	9.3	M	Abn	0.77	0.62	13.70
1100	ASD	8.7	M	Nl	1.34	0.72	32.19
1101	ASD	12.7	M	Nl	1.00	0.73	33.86
1103	ASD	9.6	F	Nl	0.92	0.72	23.01
1104	ASD	15.1	F	Nl	1.01	0.68	21.56
1105	ASD	8.1	M	Abn	1.01	0.63	14.62
1106	ASD	14.5	M	Nl	1.19	0.69	47.32
1108	ASD	10.4	M	Abn	0.93	0.62	11.87
1109	ASD	11.3	M	Nl	1.16	0.78	35.32
1112	ASD	13.5	M	Nl	1.13	0.69	35.01
1115	ASD	11.3	M	Abn	0.86	0.89	22.35
1119	ASD	16.0	M	Abn	1.07	0.66	23.96

Measurement of excitation-inhibition ratio in autism spectrum disorder using critical brain dynamics

ID	Group	Age	Gender	EEG score	EI	DFA	Rel. Power
1120	ASD	11.6	M	Nl	1.00	0.65	28.47
1121	ASD	10.7	M	Nl	1.05	0.73	29.09
1122	ASD	11.9	M	Abn	1.22	0.75	32.38
1123	ASD	7.1	F	Abn	0.91	0.59	10.66
1126	ASD	13.7	F	Abn	1.16	0.66	26.87
Typically developing children							
10011	TDC	9.6	M	Nl	1.05	0.69	27.02
10029	TDC	10.0	F	Nl	0.96	0.66	21.23
10046	TDC	12.0	F	Nl	0.88	0.67	24.61
10062	TDC	12.1	M	Nl	1.22	0.66	56.52
10077	TDC	10.5	F	Nl	0.97	0.64	16.61
10079	TDC	11.5	F	Nl	1.08	0.66	49.97
10080	TDC	12.4	F	Nl	0.76	0.62	18.76
10094	TDC	10.6	F	Nl	1.12	0.67	39.02
10095	TDC	9.3	F	Nl	0.99	0.62	29.32
10096	TDC	9.3	F	Nl	1.04	0.70	20.95
10097	TDC	11.8	F	Nl	1.02	0.66	39.93
10100	TDC	10.1	M	Nl	1.05	0.74	44.79
10101	TDC	10.0	M	Nl	1.06	0.60	15.66
10108	TDC	9.6	F	Nl	1.01	0.70	33.65
10110	TDC	10.6	F	Nl	0.95	0.61	31.39
10111	TDC	10.3	F	Nl	0.87	0.66	17.32
10117	TDC	9.6	M	Nl	1.10	0.63	21.53
10118	TDC	7.8	F	Nl	0.92	0.65	11.85
10120	TDC	11.0	M	Nl	1.04	0.56	35.49
10129	TDC	11.2	M	Nl	1.27	0.60	35.58
10131	TDC	10.8	M	Nl	0.92	0.72	21.74
10136	TDC	11.2	M	Nl	0.97	0.67	26.89
10137	TDC	7.4	F	Nl	0.92	0.68	20.12
10139	TDC	7.6	M	Nl	1.14	0.70	30.96
10140	TDC	10.5	M	Nl	1.17	0.68	25.08
10142	TDC	8.9	M	Nl	1.04	0.63	16.24
10143	TDC	8.2	M	Nl	0.96	0.69	14.10
10144	TDC	10.2	M	Nl	0.92	0.62	14.90
10147	TDC	14.5	F	Nl	0.93	0.67	27.13

Supplementary Table S1. Demographics and EEG mean biomarker values of children with ASD and typically developing children included in the study. TDC: typically developing children. M: male, F: female. EEG scores: Nl (normal), Abn (Abnormal). *fE/I* (excitation-inhibition ratio). DFA (scaling exponent of the detrended fluctuation analysis), Rel. Power (relative power).
